# Diagnosing urethral duplication including a novel radiological diagnostic algorithm

**DOI:** 10.1259/bjrcr.20150506

**Published:** 2016-12-19

**Authors:** Jonathan Frankel, Richard Sukov

**Affiliations:** Department of Radiology, Cedars Sinai Medical Center, Los Angeles, CA, USA

## Abstract

Until this 24 -year -old male was sexually active, the small dimple on his penile glans had never required any consideration. However, persistent focal irritation during sexual intercourse compelled him to pursue medical attention. Following urological consultation he was referred for imaging evaluation that demonstrated urethral duplication. After determining the subtype classification, urologic repair was successfully completed without complication. Urethral duplication is an exceedingly rare genitourinary anatomic anomaly with a variety of presentations and symptomatology. The vast majority of urethral duplications are diagnosed within the paediatric and adolescent populations. Although urethral duplication is managed by urologists, imaging identification of the particular subtype is critical for proper management planning. A thorough review of existing literature on the classification strategies and diagnostic criteria is discussed. Owing to the scarcity of clinical experience, a wide variety of diagnostic approaches are described. These often include unnecessary radiation, invasiveness and cost. A strategic and efficient diagnostic algorithm is included to guide future imaging evaluation of suspected urethral duplication. This provides the essential clinical information, while minimis ing radiation, invasiveness and cost.

## Case report

A 24-year-old previously asymptomatic male presented to his primary care physician with a complaint of focal penile irritation related to sexual intercourse. The irritation originates from a lesion along the midline on the dorsum of his penile glans. He was referred to a urologist for further evaluation.

The patient reported noticing a small dimple along the midline on the dorsum of his glans for as long as he could remember. The lesion had not previously caused him any discomfort, nor did he report a history of discharge, including urine, semen, blood or pus from the orifice. In recent months, the patient has become more sexually active and he has become aware that during and after intercourse the site is painful and inflamed for a short period of time. The patient wants to make sure there is not something wrong with his anatomy and seeks a solution.

The patient had no significant past medical history. There is no family medical history of relevance, specifically no one has reported any genital malformation. Physical examination by a urologist demonstrated a well-developed adult male. A small midline opening was present on the dorsum of the penile glans. There was no focal erythema or discharge present at the time of examination. Manipulation of the opening demonstrated a thin lumen that appeared to continue proximally along the dorsum of the penile shaft. The most likely aetiology was determined to be urethral duplication. Imaging was necessary for confirmation and to delineate the particular anatomic presentation in order to plan treatment. Demonstration of communication with the urethra or bladder would require more extensive surgical intervention to eliminate the sequelae of discharge accumulation in the lumen causing infection, cyst formation or recanalisation of the accessory orifice. The patient was referred to radiology for urethrography.

**Figure 1.**
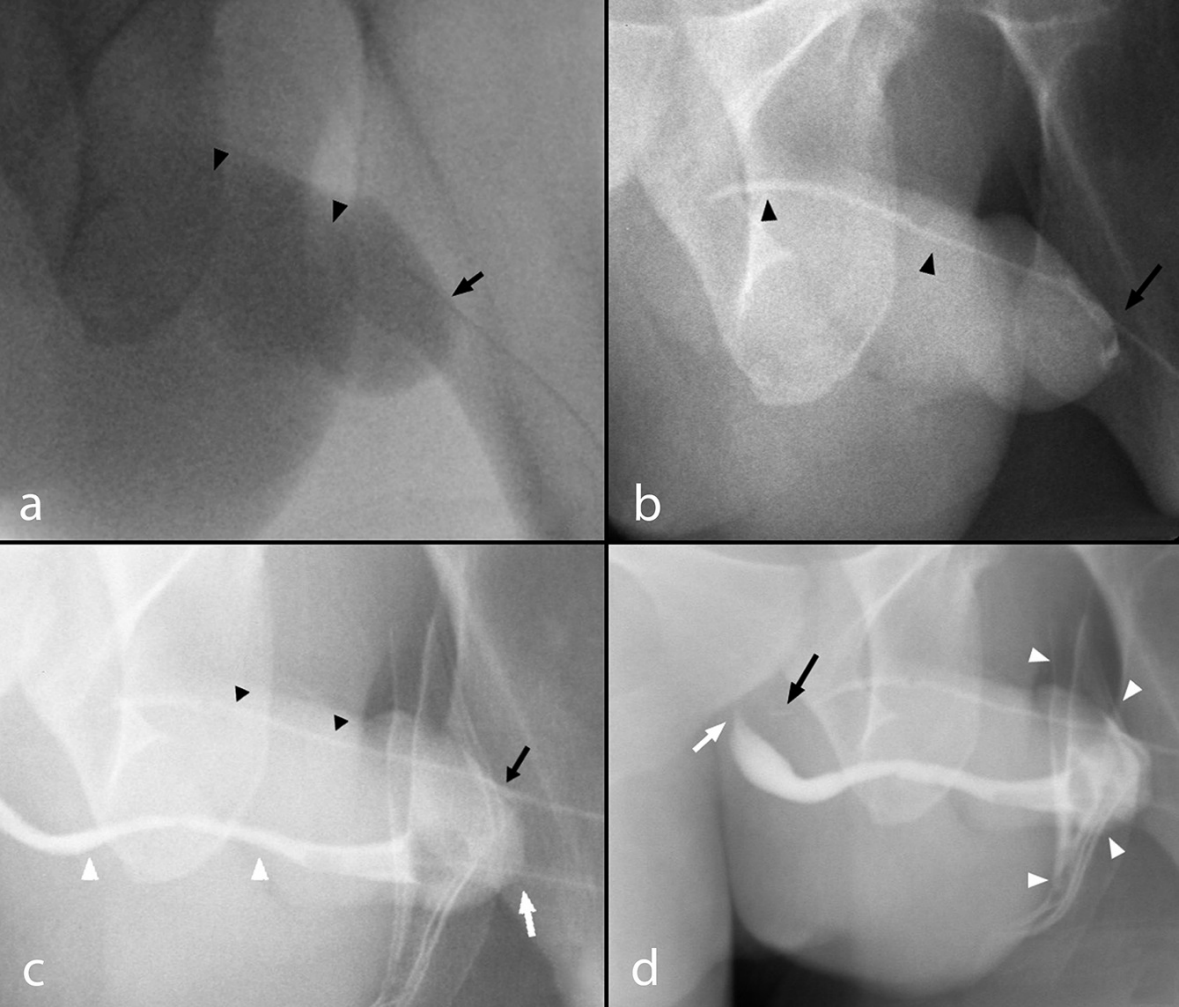
(a–d) A 23-year-old male with urethral duplication. Findings: RUG fluoroscopic coned-down spot images demonstrating (a) cannulation of the dorsal urethra meatus (black arrow) with minimal opacification of the dorsal urethra owing to resistance to Cystografin contrast flow (black arrow heads). (b) Continued gradual pressure resulted in dilatation of the atretic dorsal urethra terminating in a blind end without demonstration of communication with the ventral urethra or bladder (black arrow heads). A small volume of contrast spillage around the urethral meatus resulted owing to the significant resistance offered by the blind end (black arrow). (c) Cannulation of the ventral urethra with inflation of the Foley catheter balloon within the fossa navicularis (white arrow) and demonstration of a normal diameter ventral urethra (white arrows) relative to the atretic accessory urethra (black arrowheads). (d) Contrast is demonstrated tapering proximally at the level of the sphincteric mechanism within the ventral urethra (white arrow) despite increased injection pressure resulting in contrast spillage (white arrowheads). Persistent pressure resulted in further filling of the dorsal urethra demonstrating a tapered blind end without communication to the ventral urethra or bladder (black arrow). RUG, retrograde urethrogram.

Since no urine, semen or other discharge had ever been noted to exude from the accessory urethra, a retrograde urethrogram (RUG) was performed (Figure 1). A 5F paediatric catheter was advanced 3 cm through the dorsal accessory meatus. Simultaneously, a 6F Foley catheter was inserted into the orthotopic urethral meatus and the retention balloon inflated in the fossa navicularis. Cystografin contrast was manually instilled into both catheters. Opacification of the accessory meatus demonstrated a hypoplastic urethra traversing the dorsum of the penis and terminating blindly at the level of the levator musculature. No contrast was visualized refluxing into either the orthotopic urethra or the bladder. Contrast injected into the orthotopic urethra demonstrated a normal appearing urethra terminating at the sphincteric musculature. There was 2 mm of distance separating the terminal blind end of the accessory urethra and the orthotopic urethra. Based on the radiographic evidence a Type 1-A urethral duplication was diagnosed.

After confirming a Type 1-A urethral duplication the patient was offered and consented to operative reconstruction. A short rigid ureteroscope was used to inspect the main urethra and bladder demonstrating the appearance of normal verumontanum and sphincteric mechanisms confirming the ventral urethra as the functional urethra. The ureteroscope was then used to evaluate the accessory urethra and passed through to the blind-end. The accessory urethra was cauterized as the ureteroscope was withdrawn.

The patient tolerated the procedure well without any reported postoperative complications. At follow-up the patient did not report recurrent irritation during sexual intercourse and was pleased with the cosmetic result.

## Discussion

Although rare, urethral duplication is known to occur along a spectrum of severity distinguished by discrete anatomic features. The precise incidence is difficult to gauge as there is no consensus from the literature. There are approximately 300 known cases.^1,2^ The most common classification systems are the Effmann^3^ or Woodhouse and Williams^4^ systems. The former is considered more therapeutically practical and is more commonly used to describe anomalies. The Woodhouse and William system is not as functional but allows for subtype differentiation of the less common coronally oriented anomalies. These are not incorporated within the Effmann system and tend not to alter surgical planning.

**Figure 2.**
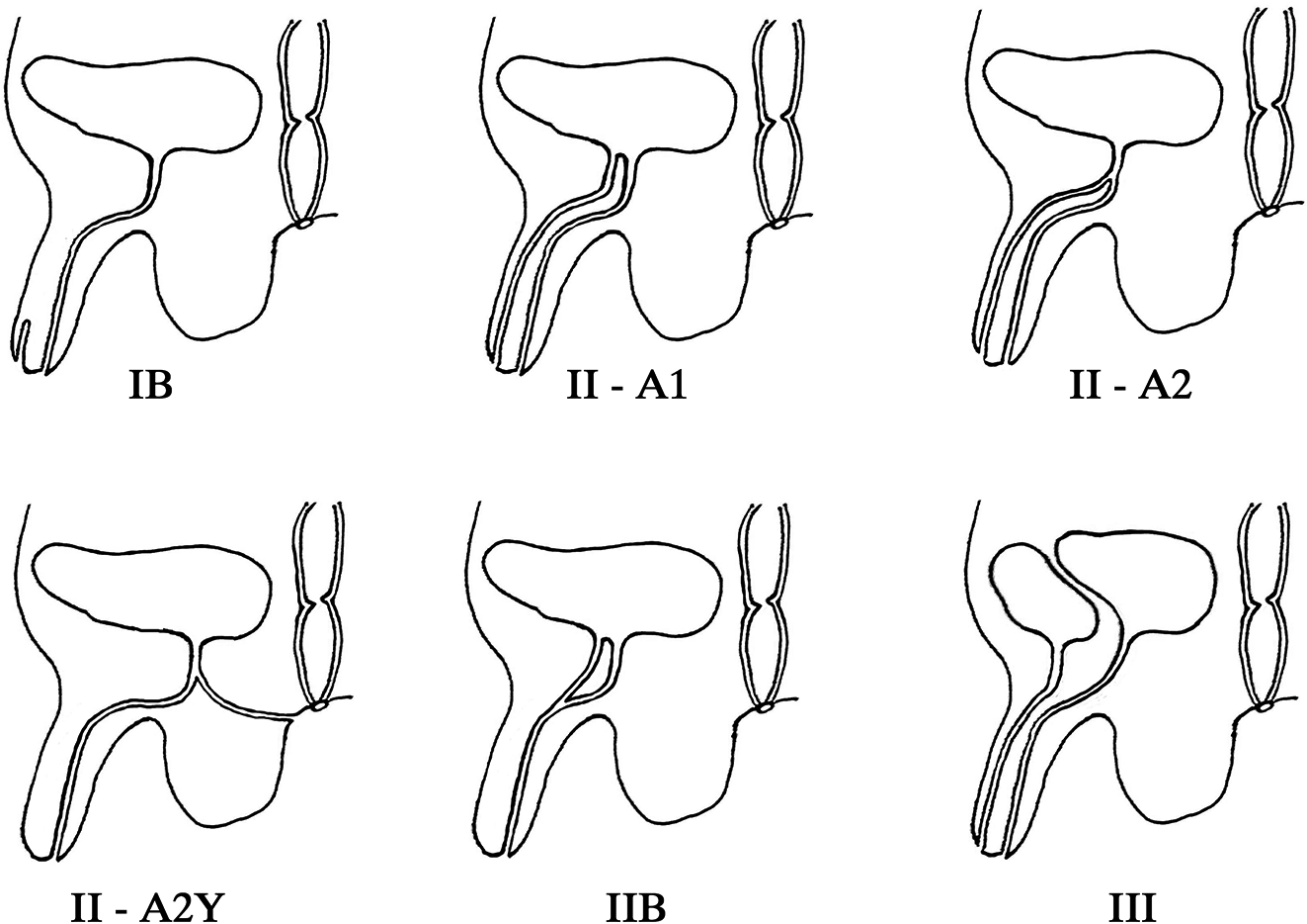
The Effmann classification system divides duplications by their therapeutic interventions and is therefore more useful for surgical planning. A blind incomplete accessory urethra is Type 1. Type 2 represents a complete functional duplication of the urethra with subtypes differentiating the origin of the duplication at either the bladder or along the orthotopic urethra. Further subtype differentiation of Type 2 duplications are determined by their site of termination; either terminating at the penile dorsum, perineum or reconnecting with the orthotopic urethra more distally in its course. Type 3 duplications are complete duplications of the bladder and functional urethra.^20^

The Effmann system classifies a blind incomplete accessory urethra as Type 1. The Type 1-A subtype is an accessory urethra that lacks communication with the functional urethra or bladder. The Type 1-B subtype is a proximal accessory urethra that derives from the functional urethra and terminates blindly within the periurethral soft tissues, similar to a urethral diverticulum. Type 2 duplications represent a complete functional duplication of the urethra with subtypes differentiating the origin of the duplication at either the bladder or along the orthotopic urethra. Further subtype differentiation of Type 2 duplications are determined by their site of termination; either terminating at the penile dorsum, perineum, rectum or reconnecting with the orthotopic urethra more distally in its course. Type 3 duplications are complete duplications of the bladder and functional urethra (Figure 2).

The aetiology of the anomalous development is uncertain and likely results from several embryologic errors.^5^ Urethral duplications occur most commonly in males; however, rare instances of female urethral duplication are described in literature.^6^ The diagnosis is most often made within the first few years of life but is usually not delayed beyond adolescence. It is uncommon for a case to be diagnosed in adulthood as in this case report. Because around 93% of urethral anomalies occur within the sagittal plane,^4^ the functional urethra, verumontanum and sphincteric mechanisms are most often located ventrally. It is a clinical imperative to ensure proper identification of the functional urethra prior to surgical intervention as the functional urethra must be preserved for proper repair and future function. ^7^

**Figure 3.**
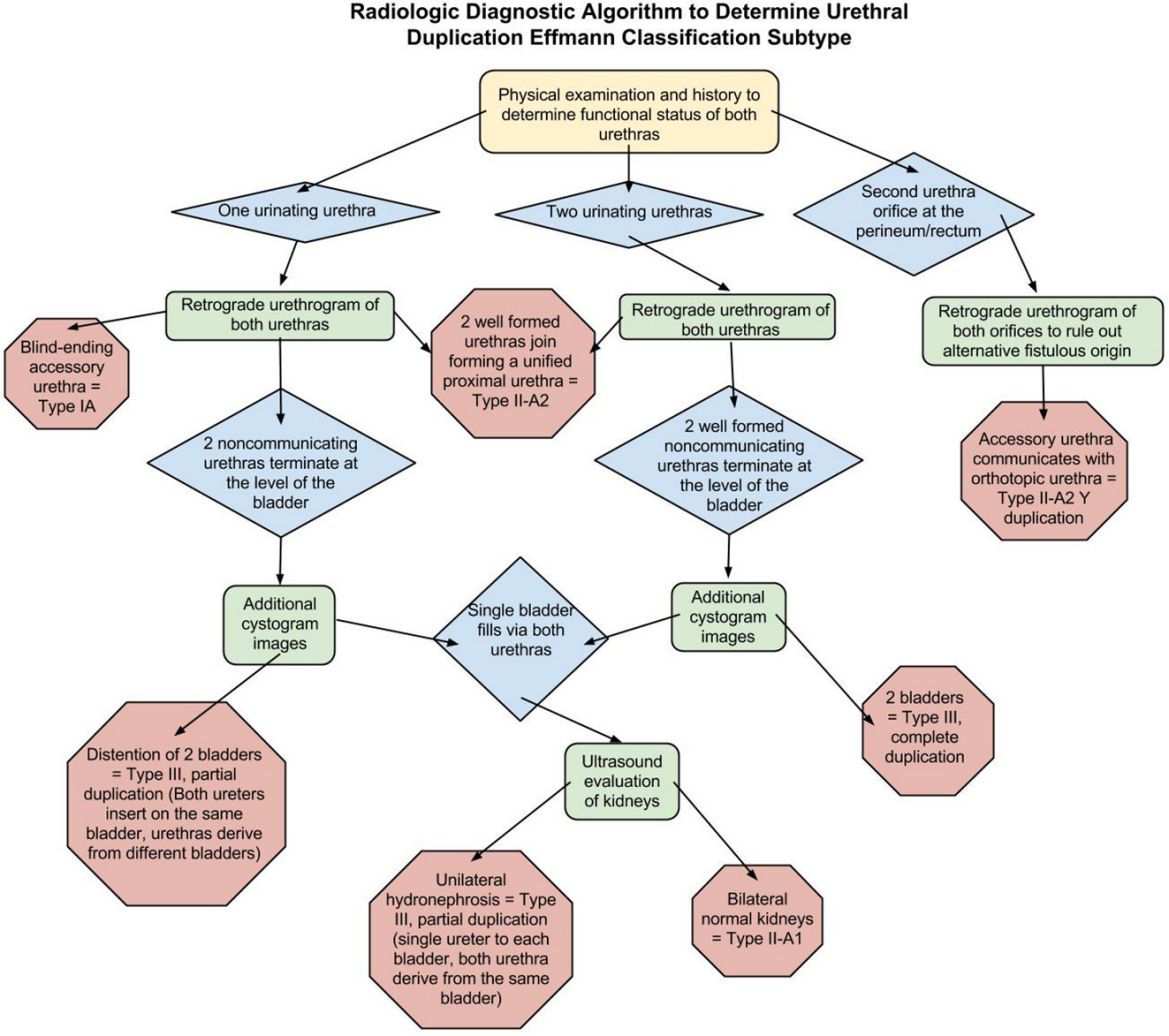
Algorithm detailing the radiological diagnostic decision tree to efficiently determine urethral duplication subtype and the functional urethra, enabling reliable clinical management utilizing the least radiation dose, invasiveness and cost. Green rectangle, diagnostic test; blue diamond, clinical finding; red octagon, definitive diagnosis.

A review of the literature reveals that a variety of methods have been employed to properly classify the anomaly and to identify the functional urethra in preparation for surgical management. Although these methods are effective in deriving the diagnosis, they often involve preventable radiation exposure, cost and invasive risk.^8,9^ No formal diagnostic strategy has been proposed to guide clinicians, surgeons and radiologists on the most efficacious diagnostic methodology to minimize radiation dosage, exposure to invasive examinations and cost. The following algorithm outlines an efficient strategy for determining the Effmann type of duplication and the functional urethra utilizing the most diagnostically informative and parsimonious methodology (Figure 3). Type 2-B duplications are excluded from the algorithm since they are cosmetically and functionally identical to normal anatomy and are usually diagnosed incidentally.^3^

The ideal initial radiologic study in all cases is a RUG. RUG allows for limited radiation exposure that can be tailored to the specific needs of the anatomy. Some forms of duplication can be diagnosed by identifying key features within the lower genitourinary tract, obviating the need for further fluoroscopic evaluation. An alternative methodology would use intravenous pyelogram (IVP), however, compared with RUG, IVP is more invasive requiring intravenous contrast as well as involve more radiation and time to evaluate the kidneys and bladder which may not be necessary for the diagnosis. Furthermore, some duplications will have a non-dominant urethra that may be obstructed with debris or present too much resistance for downstream flow to adequately opacify, ultimately requiring a follow up RUG to evaluate the urethra anatomy further increasing the radiation dose, invasiveness and time.

Initially, a thorough physical examination and history should be performed to determine the functional status of both urethras. If a second urethral orifice is identified along the perineum or rectum then Type 2-A2 Y duplication should be suspected. A RUG should be performed on both urethras to demonstrate proximal union of the urethras and to ensure the perineal/rectal orifice is not a fistulous communication of another etiology.

If only one of the urethras produces urine then a RUG of each urethra should be performed. Identification of a blind ending accessory urethra anywhere along the longitudinal length of the penis will diagnose Type 1-A. If two urethras are demonstrated with terminations approximating the location of the bladder sphincteric mechanisms, additional cystogram images should be obtained to provide additional anatomic information. If there is demonstration of two bladders then Type 3 partial duplication is present. In this case the ureters insert on the same bladder while the urethras derive from different bladders; this anomaly is usually owing to a duplicated non-functional bladder in the sagittal plane.^10^ If a single bladder distends from retrograde flow from both urethras then the patient should undergo ultrasound evaluation of the kidneys. Unilateral hydronephrosis will suggest Type 3 with partial duplication; however, in this case the ureters insert on separate bladders but the urethras derive from the same bladder resulting in outlet obstruction for the contralateral bladder and severe unilateral hydronephrosis and ultimately dysplasia of the affected kidney.^11^ If both kidneys demonstrate normal sonographic architecture then a Type 2-A1 duplication is present.

If both urethras excrete urine then Type 2 or 3 duplication is possible and a RUG should be performed on both urethras. If the proximal aspects of both urethras unify immediately distal to the bladder then the duplication is Type 2-A2. If the urethras terminate at the level of the bladder then further contrast should be administered through both urethras to opacify the bladder. If each urethra derives from a separate bladder then the duplication is Type 3 complete duplication. However, if a single bladder distends from retrograde flow from both urethras, similar to the same findings with a single voiding urethra, then the patient should undergo ultrasound evaluation of the kidneys to determine the presence of unilateral hydronephrosis to suggest a Type 3 partial duplication. If both kidneys demonstrate normal sonographic architecture then a Type 2-A1 duplication is present.

Ambiguous cases should be referred for direct cystoscopic evaluation as it is unlikely further radiographic investigations will result in more information. Direct visualization of the verumontanum via cystoscopy may be necessary in some cases where determination of the functional urethra is ambiguous.

At least 60% of urethral duplications have a second associated abnormality such as: vesicoureteral reflux, bladder exstrophy, epispadias, hypospadias, cryptorchidism, anal stenosis and renal dysplasia.^7,12–14^ Concurrent anatomic abnormalities are found more frequently in cases of bladder duplication.^4,15^ Further evaluation with cross sectional imaging of additional anomalies in the genitourinary, gastrointestinal or central nervous system may be pursued as indicated for surgical planning or clinical management. The decision to proceed with surgical intervention depends on symptom presentation and cosmetic considerations. Surgical approach depends on the anomaly type and can vary in complexity from simple meatoplasty to island flap urethroplasty, accessory urethral excision and, in the most severe cases, ureteral reimplantation.

MR urethrography is an alternative modality available for the evaluation of the lower genitourinary system. This modality, although not new, is only recently developing into a common tool for the evaluation of genitourinary pathology.^16^ Extant literature espouses the use of MR urethrography for evaluation of urethral duplication, however, in the opinion of the authors, there is insufficient evidence to support exclusive use as the primary imaging modality. A complete discussion of the benefits and limitations of MR for the evaluation of genitourinary anatomy and pathology is beyond the scope of this article, however, an appreciation of the specific features supporting or excluding its inclusion in the diagnostic algorithm is appropriate.

The diagnostic reliability of MR urethrography for evaluation of urethral anatomy is likely adequate for employing its use delineating the Effmann subtype of urethral duplication.^17^ For patients requiring sophisticated surgical planning, or if a high degree of clinical suspicion exists for associated genitourinary or gastrointestinal anomalies, MR urethrography’s additional soft tissue information would provide clinically beneficial information and an efficient means to diagnose and provide broader management information.^17,18^ In fact, MR is the best available modality for detailing the presence of anomalies, fistulas or other comorbidities that will affect surgical planning.^16^ Additionally, MR provides all of these benefits without the use of ionizing radiation.

Nonetheless, according to the opinion of the authors, the limitations of modern MR urethrography preclude its use as a practical tool in the urethral duplication diagnostic algorithm. Since most patients with urethral duplication are young children, sedation with some type of anesthesia will be necessary, increasing the invasiveness and cost of making the diagnosis. Of all potential risks associated with pediatric MR evaluation the greatest source of complication derives from anesthetic use resulting, at worst, in as many as 1:4000 deaths.^19^ Avoiding the use of anesthesia in the pediatric population receiving MR evaluation often requires the use of distraction tools such as child life consultants and sophisticated interventions that further increase the cost and duration of the study.^19^ Furthermore, the practicality of MR urethrography itself is challenging requiring the injection of gel or saline into the urethra to ensure adequate urethral distention to optimize signal strength and anatomic delineation;^16^ this increases the invasiveness of the diagnostic process as well as compromising the reliability of the results owing to under-distension or inadequate distension.

Ultimately, although exposing the patient to ionizing radiation, fluoroscopic evaluation with voiding cystourethrogramor IVP are equally if not more reliable than MR for diagnosing duplication subtype,^16^ less invasive and significantly less expensive and time consuming.

## Learning points

1. Urethral duplication is a rare congenital anomaly with a variety of presentations each necessitating reliable determination of the anatomic subtype and identification of the functional urethra to ensure proper surgical management.
2. Following the simple radiologic algorithm provided, efficacious diagnosis of urethral duplication subtype can be determined utilizing the least possible radiation, cost and invasive risk.

## Consent

Informed consent to publish this case was obtained and is held on record.
